# CAPRIN1^P512L^ causes aberrant protein aggregation and associates with early-onset ataxia

**DOI:** 10.1007/s00018-022-04544-3

**Published:** 2022-09-22

**Authors:** Andrea Delle Vedove, Janani Natarajan, Ginevra Zanni, Matthias Eckenweiler, Anixa Muiños-Bühl, Markus Storbeck, Jordina Guillén Boixet, Sabina Barresi, Simone Pizzi, Irmgard Hölker, Friederike Körber, Titus M. Franzmann, Enrico S. Bertini, Janbernd Kirschner, Simon Alberti, Marco Tartaglia, Brunhilde Wirth

**Affiliations:** 1 Institute of Human Genetics, University Hospital of Cologne, University Cologne, 50931 Cologne, Germany; 2 Center for Molecular Medicine Cologne, University of Cologne, 50931 Cologne, Germany; 3 Institute for Genetics, University of Cologne, 50674 Cologne, Germany; 4 Center for Molecular and Cellular Bioengineering, Biotechnology Center, Technische Universität Dresden, 01307 Dresden, Germany; 5 Genetics and Rare Diseases Research Division and Unit of Muscular and Neurodegenerative Disorders - the Department of Neurosciences of the Bambino Gesù Childrens’ Hospital, IRCCS, Rome, Italy; 6 Department of Neuropediatrics and Muscle Disorders, Faculty of Medicine, Medical Center—University of Freiburg, University of Freiburg, 79106 Freiburg, Germany; 7 Institute of Diagnostic and Interventional Radiology, 50937 Cologne, Germany; 8 Center for Rare Diseases, University Hospital of Cologne, 50931 Cologne, Germany

**Keywords:** Neurodegeneration, Prion-like domain, Protein misfolding, De novo variant, CRISPR/Cas9

## Abstract

CAPRIN1 is a ubiquitously expressed protein, abundant in the brain, where it regulates the transport and translation of mRNAs of genes involved in synaptic plasticity. Here we describe two unrelated children, who developed early-onset ataxia, dysarthria, cognitive decline and muscle weakness. Trio exome sequencing unraveled the identical de novo c.1535C > T (p.Pro512Leu) missense variant in *CAPRIN1*, affecting a highly conserved residue. In silico analyses predict an increased aggregation propensity of the mutated protein. Indeed, overexpressed CAPRIN1^P512L^ forms insoluble ubiquitinated aggregates, sequestrating proteins associated with neurodegenerative disorders (ATXN2, GEMIN5, SNRNP200 and SNCA). Moreover, the CAPRIN1^P512L^ mutation in isogenic iPSC-derived cortical neurons causes reduced neuronal activity and altered stress granule dynamics. Furthermore, nano-differential scanning fluorimetry reveals that CAPRIN1^P512L^ aggregation is strongly enhanced by RNA in vitro. These findings associate the gain-of-function Pro512Leu mutation to early-onset ataxia and neurodegeneration, unveiling a critical residue of CAPRIN1 and a key role of RNA–protein interactions.

**Supplementary Information:**

The online version contains supplementary material available at 10.1007/s00018-022-04544-3.

## Introduction

Cell Cycle-Associated Protein 1 (*CAPRIN1* [MIM: 601178]) is a ubiquitously expressed protein, whose levels are high in tissues characterized by an elevated cell turnover, but is also abundant in the brain [1–4]. There, it plays a crucial role as an RNA-binding protein (RBP), which regulates the transport and translation of mRNAs of synaptic proteins [2, 5]. CAPRIN1 binds to mRNAs via C-terminal RGG motifs and contains a prion-like domain (PrLD) [6, 7]. PrLDs are characterized by the lack of a defined three-dimensional structure and a low complexity sequence composition [8, 9]. They interact dynamically with other proteins and RNAs, and these interactions can trigger phase transitions as well as protein aggregation [10]. Indeed, CAPRIN1 is a component of stress granules (SGs), cytoplasmic assemblies of RBPs and stalled mRNAs that form under stress conditions [6, 11]. The N-terminal part of CAPRIN1 harbors a dimerization domain and the binding sites for the RBPs G3BP1 and FMR1 [6, 12, 13].

Interestingly, many PrLD-containing RBPs (ATXN2, TARDBP, FUS, TIA1, HNRNPA1 and HNRNPA2B1) have been associated with neurodegenerative disorders (NDs) [7]. However, missense mutations in CAPRIN1 have not been linked to neurodegeneration so far. Constitutive *Caprin1* knockout in mice causes perinatal death and impairs the formation and maintenance of synapses and neuronal network [5], while CAPRIN1 deficiency has been associated with autism spectrum disorders (ASD) and long-term memory impairment [3, 14–17].

Here we describe a novel early-onset ataxia and neurodegenerative disorder caused by a single recurrent missense variant in *CAPRIN1*. We propose a gain-of-function mechanism mediated by increased protein aggregation propensity and highlight the crucial role of RNA–protein interactions in its pathophysiology.

## Materials and methods

### Genetic studies

Genetic studies were performed as part of clinical and/or research investigations dependent on clinical presentation and family history. DNA was extracted from blood. DNA samples from A-I.1, A-I.2, A-II.3, B-I.1, B-I.2 and B-II.2 were prepared for whole exome sequencing (WES) and analyzed as described in detail in Note S1. *CAPRIN1* exon 14 genomic region was amplified by PCR following the manufacturer's protocol (Multiplex PCR Kit—QIAGEN) with specific primers (CAPRIN1-E14, Table S1).

### In silico CAPRIN1^P512L^ modeling

Human CAPRIN1 (Q14444-1) and CAPRIN1^P512L^ FASTA protein sequences were pasted in PLAAC [18]. The Relative weighting of background probabilities (α) was set to 0 and *Homo Sapiens* was selected as organism background frequency.

The CamSol intrinsic solubility profile was obtained from the CamSol web server using the same FASTA sequences [19].

### Cell culture

HEK293T cells were cultured in DMEM (ThermoFisher Scientific) supplemented with 10% FBS (SIGMA), 1% Pen Strep (ThermoFisher Scientific), 0.7 µg/ml Amphotericin B (ThermoFisher Scientific). SH-SY5Y cells were cultured in DMEM/F-12, GlutaMAX (ThermoFisher Scientific) supplemented with 10% FBS, 1% Pen Strep. All cells were cultured at 37 °C with 5% CO_2_.

iPSCs were cultures in Matrigel-coated plates (Corning) in mTeSR1 or mTeSR Plus medium (StemCell Technologies). All cells were cultured at 37 °C with 5% CO_2_.

### Constructs cloning

Total RNA was extracted from HEK293T (RNeasy Kit—QIAGEN) and reversely transcribed to cDNA (QuantiTect Reverse Transcription Kit—QIAGEN) following the manufacturer's protocol. *CAPRIN1* [GenBank: NM_005898.5] cDNA was amplified with specific primers (CAPRIN1_cl, Table S1) and cloned into pcDNA-3.1/V5-His following the manufacturer's protocol (pcDNA3.1/V5-His TOPO TA Expression Kit—Invitrogen). The c.1535C > T variant was introduced using site-directed mutagenesis with specific primers (CAPRIN1_P512L_SDM, Table S1) following the manufacturer's protocol (QuikChange II XL Site-Directed Mutagenesis Kit—Agilent Technologies). The cDNAs were then subcloned into a pEGFP-C2 vector using XhoI and BamHI restriction sites.

For the experiments in insect cells, *CAPRIN1* open reading frame optimized for insect cell expression was subcloned into pOCC468 or pOCC305 vector [20]. pOCC468-CAPRIN1 contains Twinstrep-MBP-Prescission-mGFP at the 5’ end of CAPRIN1 and Prescission-6xHIS at the 3’ end. pOCC305-CAPRIN1 contains Twinstrep-MBP-Prescission- at the 5’ of CAPRIN1 and Prescission-6xHIS at the 3’. The P512L substitution was introduced with the Q5R Site-Directed Mutagenesis Kit (New England BioLabs) using specific primers (CAPRIN1-P512L_MG, Table S1).

For the gRNA insertion in the Cas9 expression vector (pX330-hCas9-long-chimeric-grna-g2p; Leo Kurian’s laboratory), the plasmid was digested with BbsI (NEB) and annealed oligos (CAPRIN1-P512L_gRNA, Table S1) were ligated to it.

### Protein expression in HEK293T/SH-SY5Y cells

HEK293T cells were transfected with pcDNA-3.1/V5-His harboring CAPRIN1 (1–709, WT or P512L) using Lipofectamine 2000 Transfection Reagent (ThermoFisher Scientific) and samples were further processed for Solubility analysis.

SH-SY5Y cells were seeded on glass coverslips (VWR), transfected with pEGFP-C2 harboring CAPRIN1 (1–709, WT or P512L) using FuGENE HD Transfection Reagent (Promega) and processed for immunofluorescence.

### Solubility analysis

Sequential extraction of proteins from the different soluble fractions was performed following a published protocol [21]. Briefly, 24 h post transfection, cells were washed twice with PBS, lysed in cold RIPA buffer (SIGMA), and sonicated. Cell lysates were centrifuged at 100,000 g for 30 min at 4 °C to generate RIPA-soluble samples. Pellets were washed, sonicated and centrifuged twice with PBS. RIPA-insoluble pellets were then extracted with urea buffer (8 M urea, 4% CHAPS, 30 mM Tris, pH 8.5), sonicated, and centrifuged at 100,000 g for 30 min at 22 °C. Protease inhibitors were added to all buffers before use. Protein concentration was determined with the Bradford method.

### Immunoblotting

Protein lysates (10 μg) in Laemmli Buffer were heated at 95 °C for 5 min, then separated on 12% polyacrylamide gels and transferred onto nitrocellulose membranes (Merck Millipore). Membranes were blocked for 1 h in 5% BSA in TBS-T, incubated overnight with primary antibodies (Table S2) in 2.5% BSA in TBS-T at 4 °C, washed three times in TBS-T and incubated with HRP-conjugated secondary antibodies (Table S2) for 1 h at room temperature. Proteins were visualized using the Immobilon Western chemiluminescent HRP substrate (Merck Millipore). Quantification was performed with ImageLab (Bio-Rad).

### Immunofluorescence

Coverslips were washed in PBS and fixed in 4% PFA for 10 min. They were then washed three times in PBS, permeabilized in PBS-T for 10 min and blocked for 1 h at room temperature in 5% BSA in PBS-T. Coverslips were then incubated overnight with primary antibodies (Table S2) in 5% BSA in PBS-T at 4 °C, washed three times in PBS-T, incubated with conjugated secondary antibodies (Table S2) for 1 h at room temperature, washed three times in PBS-T, rinsed in water and mounted with ProLong Gold antifade reagent with DAPI (Invitrogen) on Polylysine slides (ThermoFisher Scientific). Images were acquired with a Zeiss AxioImager M2 microscope equipped with ApoTome2 system and processed using ZEN software (Zeiss). Pearson’s colocalization coefficient was obtained using the Coloc 2 tool in ImageJ. Stress granules were quantified with a self-compiled macro in ImageJ, which uses the Weka Trainable Segmentation plugin on the G3BP1 signal. A cell was considered positive if ≥ 3 signals were detected.

### CRISPR/Cas9 P512L knock-in in iPSCs

#### Homozygous CAPRIN1^P512L/P512L^ cell line

HUVEC iPSCs were CRISPR/Cas9 genetically edited into homozygous CAPRIN1^P512L/P512L^ based on a published protocol [22, 23]. Briefly, cells were seeded in mTeSR1 medium (StemCell Technologies) supplemented with 10 μM Y-27632 (Selleckchem) in a 6-wells plate at a confluency of 1.8 × 10^4^ cells/cm^2^. After 24 h cells were transfected with a Cas9 expression vector harboring a gRNA targeting CAPRIN1 exon 14 and a 400 bp ssODN (CAPRIN1-ssODN1, Table S1) using Lipofectamine 3000 Reagent (ThermoFisher Scientific). Starting 24 h post-transfection, cells were selected for 72 h with 1.5 µg/ml Puromycin (ThermoFisher Scientific). Afterwards they were split with Accutase (ThermoFisher Scientific) and seeded in mTeSR1 medium with 10 μM Y-27632 into a 96 wells plate at single-cell density. The day after, the medium was changed to mTeSR1 medium. After 12 days, colonies were split into 6-well plates and further expanded. After DNA extraction using QuickExtract DNA Extraction Solution (Lucigen), a 326 bp region targeted by the gRNA was amplified by PCR (CAPRIN1-SA, Table S1) and genome editing was screened by T7 Endonuclease (NEB) assay. In the case of a positive result, a 901 bp region (CAPRIN1-E14, Table S1) was sequenced by Sanger sequencing.

#### Heterozygous CAPRIN1^WT/P512L^ cell line

HUVEC iPSCs were CRISPR/Cas9 genetically edited into heterozygous CAPRIN1^WT/P512L^ iPSC lines following the manufacturer's protocol [24]. One hour before nucleofection, mTeSR Plus medium (StemCell Technologies) was changed with medium supplemented with 10 μM Y-27632. After detachment with Accutase, 10^6^ HUVEC iPSCs were resuspended in OptiMEM (ThermoFisher Scientific), mixed with a crRNA, tracrRNA-ATTO550, Cas9:dCas9 1:4, Electroporation enhancer, 150 bp ssODN (IDT) (CAPRIN1-ssODN2, Table S1) and nucleofected using Nucleofector 2b Device (Lonza). Cells were plated in mTeSR Plus medium with HDR enhancer (IDT). After 24 h, single cells were sorted in 96-well plates and the remaining cells in 6-well plates in mTeSR Plus medium with CloneR (StemCell Technologies) and Y-27632. Since no colonies were observed in the 96-well plates, after 5 days colonies from the 6-well plates were split again at single-cell density in a 96-well plate and subsequently expanded. Screening of the colonies was performed using an allele-specific PCR (CAPRIN1-P512L-ASP, Table S1). In the case of a positive result, a 901 bp region was sequenced by Sanger sequencing (CAPRIN1-E14, Table S1).

Off-targets for both CAPRIN1^WT/P512L^ and CAPRIN1^P512L/P512L^ lines were excluded by Sanger sequencing in the first five gRNA off-targets sites predicted using CRISPOR. Expression of pluripotency markers was confirmed by immunofluorescence.

### Neuronal differentiation

Neurons of cortical layer V and VI were generated according to a published protocol with minimal modifications [25]. In brief, iPSCs were dissociated with Accutase (ThermoFisher Scientific) and seeded on matrigel-coated (Corning) wells at a density of 3 × 10^5^/cm^2^ in mTeSR Plus medium (StemCell Technologies) supplemented with 10 μM Y-27632 (Selleckchem) (day 0—D0). On D1, the medium was replaced daily by a neural induction medium [N2/B27 (ThermoFisher Scientific), 0.5 µM LDN-193189 (Selleckchem) and 10 μM SB431542 (StemCell Technologies)]. On D8, NIM was supplemented with 20 ng/ml FGF2 (Sigma). On D9, cultures were split with Accutase and seeded in N2/B27 medium with 20 ng/ml FGF2 and 10 μM Y-27632 onto Matrigel-coated wells. On D10, N2/B27 medium with 20 ng/ml FGF2 was added. On D11, cells were cultured in N2/B27 medium with a change of medium every other day. On D19, neural precursors were dissociated and frozen. For maturation, frozen precursors were thawed and seeded in N2/B27 medium with 10 μM Y-27632, with a change of medium every other day. On D26, cells were detached with Accutase and reseeded at 5 × 10^4^/cm^2^ density on 0.05% PEI (Sigma-Aldrich) and 20 µg/ml Laminin (Sigma) coated wells/coverslips. On D27 and D29, the medium was changed to N2/B27 supplemented with 10 μM PD0325901 (Tocris) and 10 μM DAPT (Tocris). From D31 the cells were cultured in N2/B27 medium with medium changes every other day.

### Microelectrode array recordings

On D26, 7.5 × 10^4^ cells / well were seeded in a 24-well epoxy plate with a microelectrode array (MEA) (Multi Channel Systems). Cells were recorded with a Multiwell-MEA-System (Multi Channel Systems) for 3 min at 37 °C between D29 and D57 using the Multiwell-Screen software (Multi Channel Systems) and analyzed with the Multiwell-Analyser software (Multi Channel Systems). When no activity was recorded, the parameter was set to 0. The following parameters were used: 2nd order low-pass filter frequency: 3500 Hz; 2nd order high-pass filter frequency: 100 Hz; rising/falling edge of the automatic threshold estimation: 5.5/− 5.5 SD; minimum spike count in burst: 3; minimum channels participating in a network burst: 5; minimum simultaneous channels for a network burst; minimum spikes per minute: 5; minimum amplitude: 10 µV.

### SG dynamics study

On D36, iPSC-derived neurons plated onto coverslips were treated for 1 h with 0.5 mM Sodium Arsenite (SA, NaAsO_2_, Sigma-Aldrich), then washed in PBS and incubated in N2/B27 medium for 0–240 min to study SG resolution. Coverslips were then processed for Immunofluorescence.

### Recombinant protein expression and purification

mGFP-CAPRIN1 WT and P512L, as well as CAPRIN1 WT were purified from Sf9 insect cells using a baculovirus expression system [26, 27]. Cells expressing recombinant TwinstrepII-MBP-mGFP- CAPRIN1-6xHis were lysed in 50 mM Tris–HCl pH 7.5, 300 mM KCl, 150 mM Arginine-HCl, 1 mM DTT and 1 × EDTA-free protease inhibitor cocktail (Roche Applied Sciences) using a LM10 Microfluidizer (Microfluidics) at 5000 psi. The lysate was cleared by centrifugation at a maximum speed for 1 h at 4 °C. The supernatant was applied to a 5 ml Strep-Tactin®XT 4Flow® column (IBA Lifesciences GmbH) using an ÄKTA pure 25 (GE Healthcare). The column was washed with 10 column volumes (CV) of 50 mM Tris–HCl pH 7.5, 300 mM KCl, 150 mM Arginine-HCl and 1 mM DTT and the protein was eluted with 3 CV of 50 mM Tris–HCl pH 7.5, 300 mM KCl, 1 mM DTT and 50 mM biotin. The eluted protein was applied to a 5 mL HiTrap Q HP column (GE Healthcare). The column was washed with 20 CV of 50 mM Tris–HCl pH 7.5, 50 mM KCl and 1 mM DTT. Elution was achieved with a linear gradient of 20 CV of 50 mM Tris–HCl pH 7.5, 1000 mM KCl and 1 mM DTT. The elution fractions containing MBP-mGFP-CAPRIN1-6xHis were pooled and incubated 6 h at RT with 6xHis-Prescission protease (1:300 w/w) to cleave off the MBP and 6xHis tags. The sample was then applied to a HiLoad 16/600 Superdex 200 pg column (GE Healthcare) equilibrated in 50 mM Tris–HCl pH 7.5, 300 mM KCl and 1 mM DTT. mGFP-CAPRIN1 fractions were pooled, concentrated to ~ 100 µM with Amicon Ultra centrifugal 30 KDa MWCO filters (Merck Millipore), flash-frozen with liquid nitrogen and stored at − 80 °C. Purified proteins were quality controlled by SDS-PAGE and Coomassie staining. The homogeneity of GFP-tagged proteins was determined by imaging the gels using the Amersham typhoon scanner.

### Nano-differential scanning fluorimetry (nanoDSF)

mGFP-CAPRIN1 (WT or P512L) was diluted to a final protein concentration of 5 µM in 50 mM Tris/KOH pH 7.5 and 75 mM KCl. Unfolding transitions were recorded with a Prometheus Panta (Nanotemper) in high sensitivity capillaries (Nanotemper) at 0.3 °C min^−1^. Data analysis and plotting were with the R/RStudio software package.

### Total RNA and mRNA isolation

Total RNA was isolated from HeLa cells using the RNeasy Mini Kit (QIAGEN). mRNA was isolated from total RNA using the DynabeadsTM mRNA purification Kit (ThermoFisher). The tangled total RNA was prepared according to published work [20]. Poly(A) (Sigma-Aldrich, Cat#10108626001), Poly(C) (Sigma-Aldrich, Cat#P4903), Poly(G) (Sigma-Aldrich, Cat#P4404), Poly(U) (Sigma-Aldrich, Cat#P9528), and Ribosomal RNA (Bioworld, Cat#11020001-2) were all purchased. The 5’UTR-KpnB1-nanoLUC mRNA was transcribed with an mMESSAGE T7 kit (Invitrogen).

### RNA-induced aggregation of CAPRIN1

5 µM mGFP-CAPRIN1 (WT or P512L) were incubated for 4 h at RT in 25 mM HEPES/KOH pH 7.5, 75 mM, 2 mM MgCl_2_, 1% PEG-20 K with and without 50 ng/µl of RNA. When indicated, KCl concentration was increased to 500 mM or 100 µg/µl RNase A was added. Samples were mounted in 384-well plates (Greiner bio-one, #781000-06) with custom attached pegylated glass coverslips [28]. Samples were imaged with a Nikon Eclipse Ti2 inverted microscope, equipped with a Prime 95B 25 mm camera (Photometrics) and a 60x/1.2 Plan Apo Lambda water objective. Images were analyzed with Fiji.

### Fluorescence anisotropy measurements

100 nM of ATTO590-labelled RNA were mixed with increasing concentrations of the mGFP-CAPRIN1 or mGFP-CAPRIN1^P512L^, in 25 mM HEPES–KOH, pH 7.5, 75 mM KCl and incubated for 10 min at 25 °C. Fluorescence anisotropy was measured with a Tecan Spark plate reader in 384-well plates (Greiner bio-one). Fluorescent excitation was at 598 nm/20 slit width and emission was recorded at 664 nm/30 slit width. Fluorescence anisotropy was calculated using the manufacturer’s software. For RNA competition assay, x100 nM of ATTO590-labelled RNA and 3 μM of mGFP-CAPRIN1 was preincubated  for 10 min at 25 °C and then competed for with an increasing concentration of unlabeled long homopolymeric polyA RNA. The complex then competed with an increasing concentration of unlabeled long homopolymeric polyA RNA. Fluorescence anisotropy data were fitted to Eq. (1), where r is the determined fluorescence anisotropy at a given protein concentration (*c*), *L*_t_ is the ligand concentration, *K*_D_ is the apparent dissociation constant and *r*_free_ and *r*_bound_ the fluorescence anisotropy of the free and protein-complexed ATTO590-labelled RNA, respectively. Data analysis and plotting were carried out with R/RStudio software package.

1 $$r={r}_{\mathrm{free}} + \left({r}_{\mathrm{free}}-{r}_{\mathrm{bound}}\right)\cdot \frac{\frac{\left({K}_{d} +c+{L}_{t}\right) }{2}-\sqrt{{\left(\frac{\left({K}_{d}+c+{L}_{t}\right)}{2}\right)}^{2}-4c{L}_{t}}}{{L}_{t}}$$

### FCS measurements

FCS measurements were carried out using a LSM780 (Zeiss) confocal microscope. The system and measurements were calibrated using ATTO488 (*D* = 4.0 ± 0.1 × 10^–6^ cm^2^ s^−1^ at 25 °C) [29]. Diffusion times were recorded using a 488 nm argon laser for excitation and a 495–555 nm bandpass filter for emission, at 3.5 μW laser power, AOTF dampening factor of 10% with 15 rep for 10 s. The diffusion coefficients were calculated using Eq. (2).

2 $$D = {\omega }^{2}/4{\tau }_{D}$$

The radius of the proteins was then calculated using the Stokes–Einstein Eq. (3).

3 $$D = {k}_{b}T/6\pi \eta {R}_{h}$$

All FCS measurements were carried out with 50 nM of mGFP-CAPRIN1 WT or P512L or mGFP in 25 mM Tris-KOH, pH 7.5, 75 mM KCl and 1 mM DTT at 22 °C. For oligomerization experiments 50 nM mGFP-CAPRIN1 WT were mixed with either 100 or 2000 nM unlabelled CAPRIN1. ATTO488 dye and mGFP were used as standards to calculate the diffusion volume and the number of GFP molecules in the diffusion volume respectively. Data analysis and plotting were carried out with R/RStudio software package.

### Statistical analysis

All statistical analyses were performed using the software Prism 9 (GraphPad). Unpaired *t* test were used for the comparison of two groups (solubility analysis, aggregates number/size, circularity, colocalization, biochemical properties), while one-way ANOVA was used for the comparison of three groups. Chi-square test was applied to compare frequencies of aggregates in transfected SH-SY5Y cells.

## Results

### Two independent ataxic individuals carry the same de novo Pro512Leu mutation in CAPRIN1

**Fig. 1:**
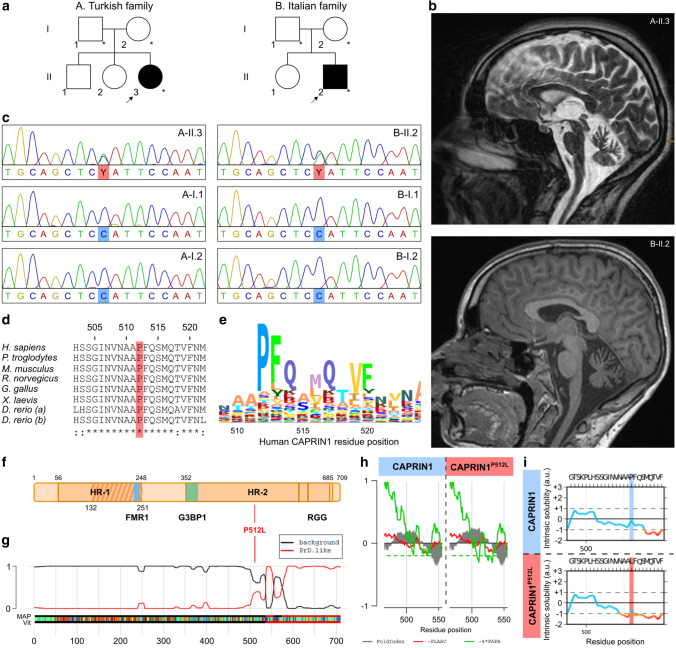
Clinical and genetic characterization of the patients carrying the CAPRIN1 mutation. **a** Pedigrees of the families. Individuals marked with asterisks underwent WES. **b** Cerebellar and cerebral atrophy in the affected individuals. Sagittal brain MRI section of affected individuals A-II.3 and B.II-2 at 16 and 12 years of age, respectively. **c** Sanger sequencing of the families’ individuals. Pherograms confirm the heterozygous de novo c.1535C > T variant in *CAPRIN1* in the affected individuals and its absence in the unaffected parents. **d** CAPRIN1 Pro512 residue is highly conserved. Protein sequence alignment of CAPRIN1 orthologues displays high conservation in the region of the P512L mutation (red). **e** HMM logo of CAPRIN1 protein sequence alignment confirms the high conservation of the Pro512 residue. **f** Schematic representation of CAPRIN1. Highlighted are: homology region 1 and 2 (HR-1, residues 56-248; HR-2, residues 352-685) with CAPRIN2; CAPRIN1 dimerization region (residues 132-251, //) [12]; FMR1 binding region (residues 231-245, blue) [13]; G3BP1 binding region (residues 352-380, green) [6]; RGG motifs (RGG) [1]. **g** CAPRIN1 C-terminal region is a PrLD. PLAAC application predicts a PrLD between residues 537 – 709. The position of the CAPRIN1^P512L^ mutation is highlighted in red. **h** PLAAC in silico modeling of the P512L mutation. The CAPRIN1^P512L^ mutation lowers the -4*PAPA score (green solid line), crossing the cutoff (green dashed line) and indicating an increased aggregation propensity. **i** CamSol in silico modeling of the CAPRIN1^P512L^ mutation. The CAPRIN1^P512L^ mutation lowers CAPRIN1 solubility

Affected individual II.3 of family A was referred for genetic counseling at the age of 10 years because of the development of gait abnormalities and predominantly proximal muscle weakness (positive Gower’s sign). She was born to non-consanguineous parents of Turkish descent and had two older healthy siblings (Fig. 1a). Her symptoms worsened over the following years, with increased muscle weakness and the development of ataxia with light tremor and dysdiadochokinesis. The progressive trunk instability and scoliosis lead her to be first confined to a wheelchair and later to a bed. The motor deficits were accompanied by bulbar symptoms (dysphagia and dysarthria), deficits in sustained attention, social withdrawal and cognitive decline. She attended a mainstream school but later changed to a special needs school. Formal testing revealed an intelligence quotient of 64. Standard laboratory and metabolic tests were negative. Electromyography and nerve conduction studies identified a sensorimotor axonal neuropathy. Muscle biopsy revealed neurogenic fiber atrophy and suralis nerve biopsy uncovered a chronic axonal neuropathy with loss of small- and big-caliber nerve fibers. Magnetic resonance imaging (MRI) at 16 years of age displayed cerebral and cerebellar atrophy (Fig. 1b). Deletions of *SMN1* were excluded and no causative variant was found using an in-house NGS gene panel covering 62 genes associated with lower motor neuron disorders [30].

Affected individual II.2 of family B was born to non-consanguineous parents of Italian descent and had a healthy sister (Fig. 1a). He articulated his first words with slight phonetic problems and presented with dysarthria at the age of 4 years. At the age of 7 years, he developed slowly progressive ataxia and learning difficulties (IQ: 77). By the age of 11 years, his trunk stability worsened and standing up became more difficult. At the age of 12 years, MRI showed global cerebellar atrophy (Fig. 1b). At the age of 13 years, he showed increased muscle fatigue and muscle hypotrophy, with absent deep tendon reflexes in all four limbs. He also became increasingly anxious but improved with psychotherapy. Standard laboratory and metabolic tests were negative. Somatosensory evoked potentials (SSEPs) were reduced in the lower limbs.

Both affected individuals and the respective parents (A-I.1, A-I.2, A-II.3, B-I.1, B-I.2, B-II.2) were subjected to trio whole exome sequencing. Variant filtering followed standard metrics (quality, allele frequency < 0.01 in gnomAD; Note S1). Variants were prioritized assuming an autosomal recessive model of inheritance or a de novo mutation occurrence. The c.1535C > T (NC_000011.10:g.34090659C > T, exon 14, p.Pro512Leu) *CAPRIN1* (GenBank: NM_005898.5) variant resulted as the most likely candidate in both families: this variant was absent in gnomAD [31], affects a highly conserved residue and was predicted to be deleterious by multiple scores (CADD PHRED score: 29.8; SIFT: 0 [deleterious]; PolyPhen-2: 0.993 [probably damaging]) (Fig. 1c–e) [32, 33]. In addition, the likelihood that the same de novo variant occurs in two independent individuals is extremely rare (1 in 154 million, Note S2).

Several other characteristics support the involvement of *CAPRIN1* as a neurodegeneration-causing gene: (i) *CAPRIN1* is highly expressed in the human and murine central nervous system, and in particular in the cortex and cerebellum [3, 4]; (ii) CAPRIN1 is an RBP harboring a PrLD (Fig. 1f and g) [2, 7], feature shared by many ND-linked genes [10]; (iii) CAPRIN1 is a component of SGs [6], whose role in NDs is well documented [34]; CAPRIN1 is an interacting partner of ATXN2 and GEMIN5 [35, 36], whose pathogenic variants are associated with ataxia [37, 38], and FMR1 (Fig. 1f) [13], a protein associated with fragile X tremor/ataxia syndrome (FXTAS) [39].

Note added in proof: Just after the acceptance of our manuscript, we were notified by GeneMatcher of a patient identified at NINDS, NIH (female, 14 yrs old) with exactly the same de-novo variant and an identical phenotype (cerebellar atrophy, ataxia and motor > sensory axonal neuropathy) as the other two patients (S. Donkervoort and C.G. Bönnemann, direct communication).

### In silico CAPRIN1^P512L^ modeling predicts increased aggregation propensity

Since mutations linked to NDs in PrLD-containing proteins cause increased protein misfolding and the P512L substitution occurs close to the PrLD (Fig. 1f) (residues 537–709) [20, 40–43], we hypothesized that it would render CAPRIN1 prone to misfolding and aggregation. Indeed, in silico analysis of CAPRIN1 and CAPRIN1^P512L^ using the PLAAC, CamSol and ZipperDB tools predicted an increase in aggregation propensity and an increase in protein insolubility for CAPRIN1^P512L^ (Fig. 1h, i and S1a) [18, 19, 44].

### CAPRIN1^P512L^ forms insoluble aggregates

**Fig. 2:**
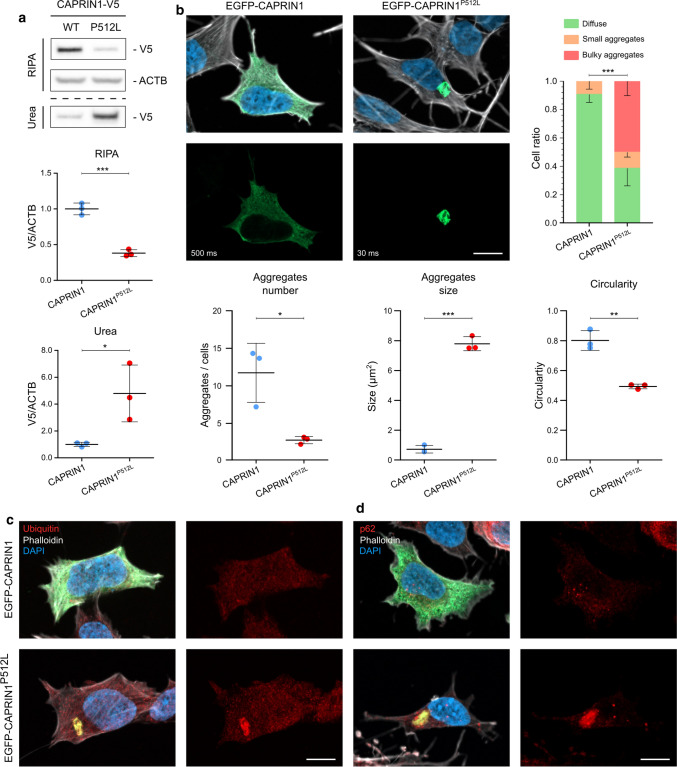
CAPRIN1^P512L^ is less soluble than CAPRIN1. **a** Immunoblot from sequential protein extraction with RIPA and urea buffers in HEK293T transfected cells. While CAPRIN1-V5 is mostly eluted in the RIPA fraction, CAPRIN1^P512L^-V5 is more insoluble and is eluted in the urea fraction (Bars: mean ± SD; *n* = 3; unpaired *t* test: ***p < 0.001, *p < 0.05). **b** CAPRIN1^P512L^ forms aggregates. SH-SY5Y cells transiently expressing EGFP, EGFP-CAPRIN1 and EGFP-CAPRIN1^P512L^. While EGFP-CAPRIN1 mostly shows a diffuse cytoplasmatic distribution, EGFP-CAPRIN1^P512L^ forms few, bulky aggregates. The exposure time for the green channel is reported in the panel. (Scale bar: 10 µm. Bars: mean ± SD; *n* ≥ 3; *χ*^2^ test: ***p < 0.0001; unpaired *t* test: *p < 0.05; **p < 0.01; ***p < 0.001). **c** CAPRIN1^P512L^ aggregates are positive for Ubiquitin. **d** CAPRIN1.^P512L^ aggregates are positive for p62. (Scale bar: 10 µm)

To investigate the potentially increased aggregation propensity of CAPRIN1^P512L^, we overexpressed V5-tagged CAPRIN1 and CAPRIN1^P512L^ in HEK293T cells and sequentially extracted proteins from a more soluble (RIPA) and less soluble (urea) fraction. CAPRIN1-V5 was mainly eluted in the RIPA-soluble fraction, while CAPRIN1^P512L^-V5 exhibited reduced solubility and was recovered in the urea-soluble fraction (Fig. 2a), a behavior found in other mutant PrLD-containing proteins related with degenerative disorders [21].

**Fig. 3:**
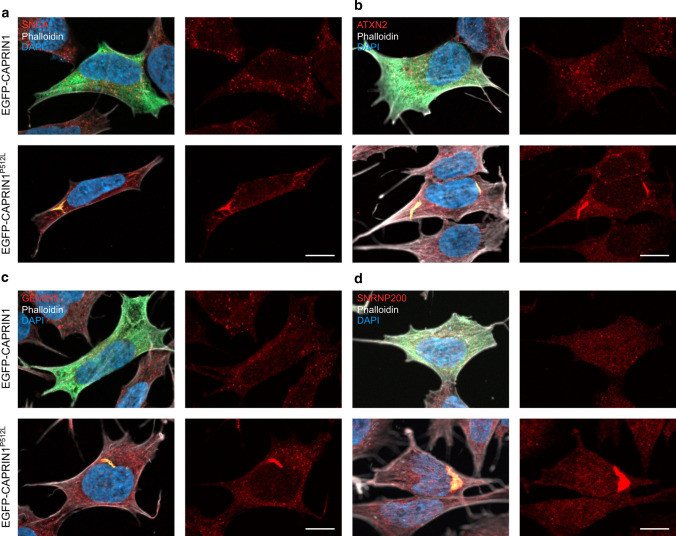
CAPRIN1^P512L^ aggregates are positive for NDs-related proteins. **a** EGFP-CAPRIN1^P512L^ aggregates are positive for SCNA. **b** EGFP-CAPRIN1^P512L^ aggregates are positive for ATXN2. **c** EGFP-CAPRIN1^P512L^ aggregates are positive for GEMIN5. **d** EGFP-CAPRIN1^P512L^ aggregates are positive for SNRNP200. (Scale bar: 10 µm)

Remarkably, in SH-SY5Y cells, overexpression of EGFP-tagged CAPRIN1 mostly revealed diffuse cytoplasmic localization and coalesces in small round clusters (Fig. 2b and S1b), compatible with the induction stress granules, as previously reported [2, 6]. On the contrary, EGFP-CAPRIN1^P512L^ mostly formed a few, large aggregates (Fig. 2b–d, 3a–d and S1b–d).

### CAPRIN1^P512L^ aggregates are positive for typical NDs markers

Since protein misfolding and impairment of the protein quality control (PQC) are widely recognized pathomechanism of NDs [45], we investigated protein homeostasis markers, such as ubiquitin and p62. Under physiological conditions, the formation of aggregates is prevented by the activity of molecular chaperons of the PQC, which are also able to unfold misfolded proteins. When folding is not possible, misfolded proteins are ubiquitinated by E3 ubiquitin ligases and directed to proteasomal degradation via the ubiquitin–proteasome system (UPS) [46, 47]. Indeed, bulky CAPRIN1^P512L^ aggregates were positive for ubiquitin (Fig. 2c). Moreover, since insoluble aggregates can inhibit the 26S proteasome and be targeted for lysosomal degradation by macroautophagy [48, 49], we stained CAPRIN1^P512L^ aggregates for p62/SQSTM1 positivity and we could indeed detect a strong signal, as reported for other NDs (Fig. 2d) [50]. Taken together, these results suggest that CAPRIN1^P512L^ misfolds and becomes targeted for degradation.

### CAPRIN1^P512L^ aggregates sequester ataxia-related proteins

We next investigated if CAPRIN1^P512L^ inclusions sequester other proteins, resembling the pathophysiology of other age-related neurodegenerative disorders: for example, α-synuclein (SNCA) inclusions (Lewy bodies) can be found in Parkinson’s disease (PD) and Lewy bodies dementia (LBD) [51]. Likewise, TARDBP aggregates are a common feature in both amyotrophic lateral sclerosis (ALS) and frontotemporal lobar degeneration (FTLD) pathology despite their genetic heterogeneity [52, 53]. CAPRIN1 has also been detected in aggregates in TARDBP or FUS-positive ALS spinal cord motor neurons [35, 54, 55]. We detected a strong SNCA positivity for CAPRIN1^P512L^ aggregates (Fig. 3a, Table 1), but no colocalization with TARDBP and FUS (Fig. S1c and S1d).

**Table 1:** Pearson’s correlation coefficients for colocalization

	CAPRIN1	CAPRIN1^P512L^	*p* value
Ubiquitin	0.07 ± 0.09	0.71 ± 0.17	***
p62	0.39 ± 0.09	0.69 ± 0.17	**
SNCA	0.34 ± 0.14	0.89 ± 0.12	***
ATXN2	0.43 ± 0.07	0.75 ± 0.02	***
GEMIN5	0.40 ± 0.07	0.81 ± 0.22	**
SNRNP200	0.11 ± 0.06	0.94 ± 0.03	***

*p < 0.05, **p < 0.01, ***p < 0.001

We next examined whether CAPRIN1^P512L^ aggregates would sequester other known CAPRIN1 binding partners. We focused on specific candidates: ATXN2, whose polyQ expansions cause spinocerebellar ataxia 2 (SCA2) [37], and GEMIN5, whose biallelic mutations cause neurodevelopmental delay and ataxia [38]. Indeed, both proteins were present in the CAPRIN1^P512L^ inclusions (Fig. 3b and c; Table 1).

Due to the progressive muscle atrophy of the affected individuals, we additionally investigated if the aggregates contained SNRNP200, another CAPRIN1 interacting partner that has been reported in the cortical and spinal motor neurons of ALS cases and indeed we could detect it (Fig. 3d; Table 1) [36]. Taken together, these results indicate that CAPRIN1^P512L^ inclusions are able to sequester multiple ND and ataxia-related proteins.

### CAPRIN1^P512L^ iPSC-derived neurons show reduced neuronal activity

**Fig. 4:**
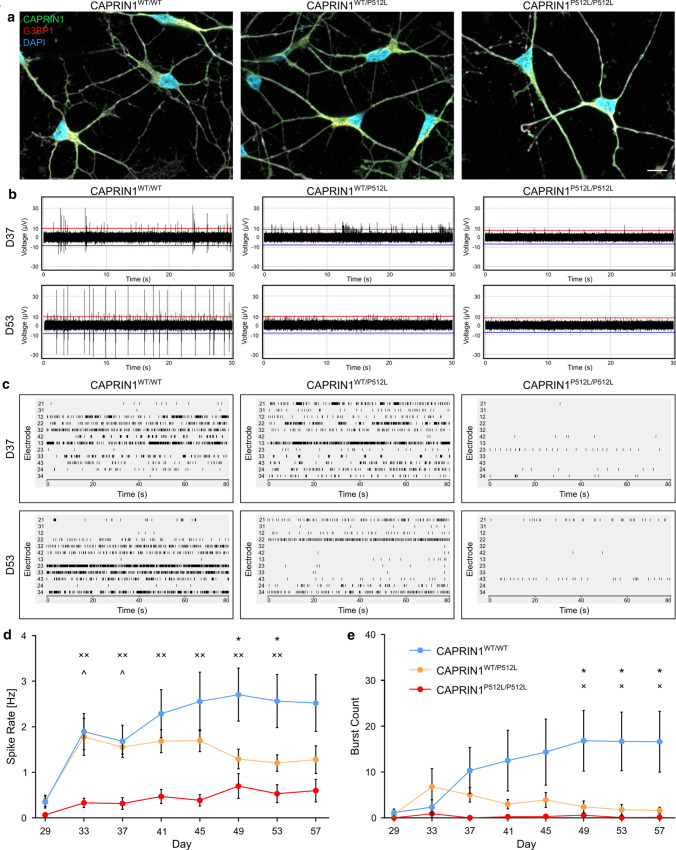
CAPRIN1^P512L^ neurons show reduced neuronal activity. **a** At maturation (D36), CAPRIN1^WT/P512L^ and CAPRIN1^P512L/P512L^ do not show overt protein aggregation. **b** Representative traces from a MEA electrode at day 37 (D37) and 53 (D53). **c** Representative activity plots of a MEA well at day 37 (D37) and 53 (D53). **d** CAPRIN1^WT/P512L^ and CAPRIN1^P512L/P512L^ iPSC-derived neurons show reduced spike rate in comparison with CAPRIN1^WT/WT^ ones (Bars: mean ± SEM; n = 3; one-way ANOVA: *CAPRIN1^WT/WT^ vs CAPRIN1^WT/P512L^, ˟CAPRIN1^WT/WT^ vs CAPRIN1^P512L/P512L^, ^CAPRIN1^WT/P512L^ vs CAPRIN1^P512L/P512L^, */˟/^p < 0.05, ˟˟*p* < 0.01). **e** CAPRIN1^WT/P512L^ and CAPRIN1^P512L/P512L^ iPSC-derived neurons show reduced burst counts in comparison with CAPRIN1^WT/WT^ ones (Bars: mean ± SEM; *n* = 3; one-way ANOVA: *CAPRIN1^WT/WT^ vs CAPRIN1^WT/P512L^, ˟CAPRIN1^WT/WT^ vs CAPRIN1^P512L/P512L^, ^CAPRIN1^WT/P512L^ vs CAPRIN1^P512L/P512L^, */˟/^*p* < 0.05)

To study the effects of the P512L substitution in a human neuronal cell model, we generated the heterozygous CAPRIN1^WT/P512L^ and the homozygous CAPRIN1^P512L/P512L^ isogenic cell lines from the CAPRIN1^WT/WT^ HUVEC iPSC line using CRISPR/Cas9 genome editing (Fig. S2a). We then differentiated them in cortical neurons using the protocol from Schuster et al. 2020 (Fig. S2b and S2c). These iPSC-derived neurons do not show any significant change in CAPRIN1 levels at neuronal maturation (D36), nor any overt morphological alteration of the cell soma or the neurites (Fig. 4a).

In particular, both iPSCs and iPSC-derived neurons harboring the CAPRIN1^P512L^ mutation did not display any protein aggregates, even upon proteasomal inhibition (Fig. S3a-b and S4a). Their absence, however, is reported for many other iPSC-derived neuronal cells lines that harbor mutations associated with protein aggregation in tissue sections from individuals suffering of ataxia, Parkinson’s disease or Huntington’s disease (HD) [56–58].

Since in several disease models electrophysiological changes in neurons precede neuronal loss [59], we recorded the spontaneous neuronal activity using a microelectrode array system. Interestingly, while CAPRIN1^WT/WT^ and CAPRIN1^WT/P512L^ neurons increased their firing rate upon maturation, CAPRIN1^P512L/P512L^ neurons showed a clearly reduced spike rate and almost no bursting throughout the whole recording period (Fig. 4b–e). On the other hand, after an initial overlap, also the activity of CAPRIN1^WT/P512L^ neurons progressively decreased (Fig. 4b–e).

### CAPRIN1^P512L^ iPSC-derived neurons show impaired stress granules dynamics

**Fig. 5:**
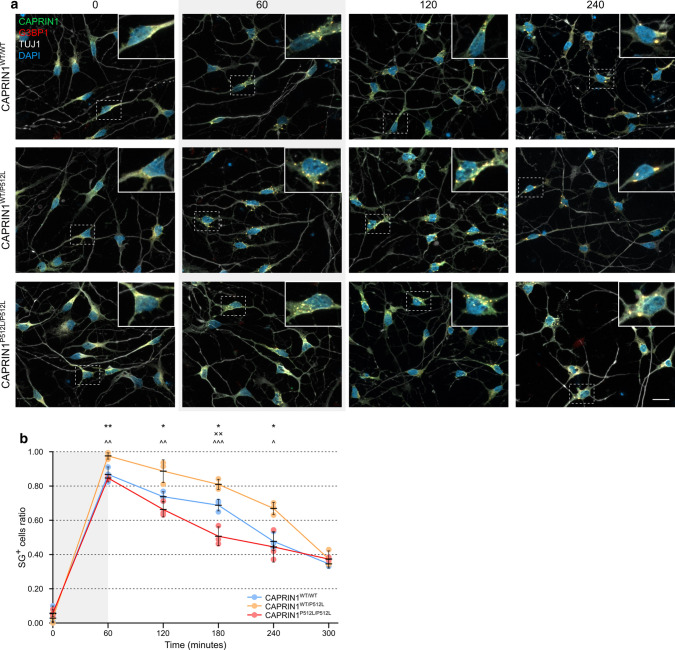
CAPRIN1^P512L^ neurons show impaired stress granules dynamics. **a** Representative pictures of the neurons. iPSC-derived neurons were treated for 60 min with 0.5 mM NaAsO_2_. Medium was then exchanged with normal medium and cells were incubated at different time points and fixed. The number on top indicate the time in minutes. **b** Quantification of the SG^+^ cell ratio (Bars: mean ± SEM; n = 3; one-way ANOVA: *CAPRIN1^WT/WT^ vs CAPRIN1^WT/P512L^, ˟CAPRIN1^WT/WT^ vs CAPRIN1^P512L/P512L^, ^CAPRIN1^WT/P512L^ vs CAPRIN1^P512L/P512L^, */˟/^*p* < 0.05; **/˟˟/^^*p* < 0.01; ^^^*p* < 0.001. Scale bar: 20 µm)

Since CAPRIN1 represents one of the main components of SGs [6, 11], and disease-linked mutations in TARDBP, FUS or C9ORF72 cause an increase of cells presenting SGs upon stress [60–62], we hypothesized that the CAPRIN1^P512L^ mutation could alter their dynamics. Therefore, we treated the iPSC-derived neurons with sodium arsenite (SA), a common SG inducer [6], and studied their resolution at different time points. Intriguingly, upon SA treatment, a higher fraction of CAPRIN1^WT/P512L^ neurons showed SGs in comparison to both CAPRIN1^WT/WT^ and CAPRIN1^P512L/P512L^ neurons (Fig. 5a and b). Moreover, in CAPRIN1^WT/P512L^ neurons the resolution of the SGs occurred slower than in the other cell lines, resulting in the persistence of SG for a longer time after stress removal. Strikingly, this difference could not be observed in CAPRIN1^P512L/P512L^ neurons, where the SGs resolution tended to be even faster than in the CAPRIN1^WT/WT^ neurons, suggesting a more complex scenario where the CAPRIN1 properties and interactions might play a major role.

### CAPRIN1^P512L^ adopts an extended conformation

**Fig. 6:**
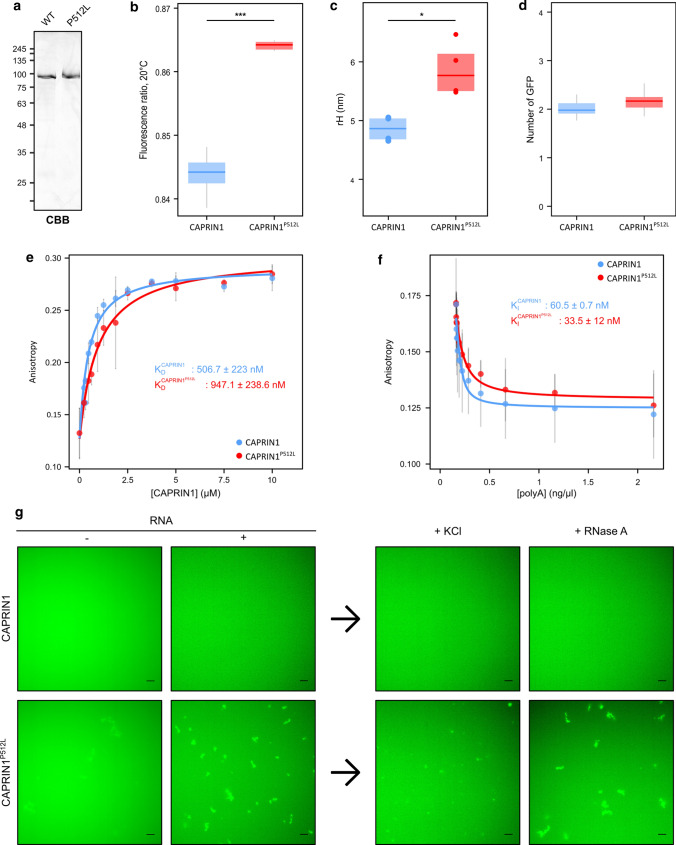
Dynamics of CAPRIN1^P512L^ and RNA. **a** Purified mGFP-CAPRIN1 and mGFP-CAPRIN1^P512L^ stained with Coomassie (CBB) on SDS-PAGE. **b** CAPRIN1^P512L^ adopts a more extended conformation than CAPRIN1. The fluorescence ratio (F350/F330) at 20 °C of the two proteins is shown (mGFP-CAPRIN1: 0.845 ± 0.006; mGFP-CAPRIN1^P512L^: 0.864 ± 0.009; *n* = 9; unpaired *t* test: ***p < 0.001). **c** CAPRIN1^P512L^ adopts a more extended conformation than CAPRIN1. Hydrodynamic radii (rH) were calculated using FCS (mGFP-CAPRIN1^P512L^: 4.8 ± 0.5 nm; mGFP-CAPRIN1^P512L^: 5.8 ± 0.2 nm; *n* = 3; unpaired *t* test: *p* < 0.05). **d** CAPRIN1^P512L^ dimerization is not impaired. The GFP brightness comparison of 50 nM mGFP-CAPRIN1 and mGFP-CAPRIN1^P512L^ to mGFP indicate that both proteins form dimers. **e** CAPRIN1^P512L^ has a reduced RNA affinity. Fluorescence anisotropy measured the binding affinity of CAPRIN1 and CAPRIN1^P512L^ using ^ATTO590^ssRNA. (K_D_^CAPRIN1^: 506 ± 223 nM; K_D_^CAPRIN1−P512L^: ~ 947 ± 238.6 nM; *n* = 3). **f** CAPRIN1^P512L^ has a reduced RNA affinity. An increasing amount of unlabeled polyA was added to ^ATTO590^ssRNA-bound mGFP-CAPRIN1 or mGFP-CAPRIN1^P512L^ and changes in anisotropy were measured (K_I_^CAPRIN1^: 60.5 ± 0.7 ng/µl; K_I_^CAPRIN1−P512L^: 33.5 ± 12 ng/µl; *n* = 2). **g** CAPRIN1^P512L^ aggregation is enhanced by RNA incubation. Upon RNA addition, mGFP-CAPRIN1^P512L^ aggregates while mGFP-CAPRIN1 remains soluble. KCL or RNase A were added to check the reversibility of the interaction (Scale bar: 10 µm)

To investigate whether the P512L mutation influences CAPRIN1 structure, we characterized recombinantly produced and purified mGFP-CAPRIN1 and mGFP-CAPRIN1^P512L^ (Fig. 6a). We used nano-differential scanning fluorimetry (nanoDSF) to monitor the tertiary structure and unfolding transitions of CAPRIN1. This revealed significant differences in the fluorescence ratio (F350/F330) at 20 °C and increased stability of mGFP-CAPRIN1^P512L^ in comparison to mGFP-CAPRIN1 (Fig. 6b, Table 2). These data suggest that the mutation does not cause a substantial destabilization of the protein and that the two proteins have a similar tertiary structure. Dynamic light scattering (DLS) and fluorescence correlation spectroscopy (FCS) measurements showed that mGFP-CAPRIN1^P512L^ exhibits an increased hydrodynamic radius compared to that of mGFP-CAPRIN1 (Fig. 6c, Table 2). Taken together, the data suggest that CAPRIN1^P512L^ adopts an extended yet near-native conformation.

**Table 2:** Biochemical properties of mGFP-CAPRIN1 and mGFP-CAPRIN1^P512L^

	CAPRIN1	CAPRIN1^P512L^	*p* value
Ratio, 20 °C (F350/330)^a^	0.84 ± 0.006	0.86 ± 0.009	**
*T*_m_ (°C)^b^	47.8 ± 0.1	48.4 ± 0.1	***
Radius (FCS, nM)	4.8 ± 0.5	5.8 ± 0.2	*

*p < 0.05, **p < 0.01, ***p < 0.001

^a^F350/F330 fluorescence ratio at 20 °C

^b^Unfolding temperature

### The P512L mutation does not impair CAPRIN1 dimerization

Given the differences in hydrodynamic radius between CAPRIN1 and CAPRIN1^P512L^ and the ability of CAPRIN1 to form dimers [12], we used FCS to test for changes in CAPRIN1 oligomerization. We measured the brightness of mGFP-CAPRIN1 and mGFP-CAPRIN1^P512L^ and compared it to the brightness of free GFP. Both proteins were shown to associate into dimers in solution even at concentrations as low as 50 nM (Fig. 6d). Consistent with the formation of CAPRIN1 dimers, the GFP brightness decreased when mGFP-CAPRIN1 was mixed with an excess of unlabeled CAPRIN1, demonstrating the formation of spectroscopic heterodimers (Fig. S5a). In accordance with the distance between the mutated residue and the annotated dimerization domain (residues 132–251) [12], our data demonstrate that the P512L mutation does not affect dimerization, but rather results in an expanded conformation of the protein.

### CAPRIN1^P512L^ aggregation is enhanced by RNA

Since CAPRIN1 is an RBP, we examined whether this conformational change would alter its affinity for RNA. To this end, we incubated CAPRIN1 with ATTO590-labelled single-stranded RNA (ssRNA). CAPRIN1^P512L^ showed reduced RNA affinity (K_D_^CAPRIN1^: ~ 506 ± 223 nM; K_D_^CAPRIN1−P512L^: ~ 947 ± 239 nM; Fig. 6e). We then tested the reversibility of the CAPRIN1-RNA interaction by adding unlabeled long homopolymeric polyA RNA as a competitor. In accordance with the previous data, CAPRIN1 binds ssRNA ~ twofold tighter than CAPRIN1^P512L^ (K_I_^CAPRIN1^: 60.5 ± 0.7 ng/µl; K_I_^CAPRIN1−P512L^: 33.5 ± 12 ng/µl; Fig. 6f).

To further investigate CAPRIN1^P512L^ properties, we observed mGFP-CAPRIN1 and mGFP-CAPRIN1^P512L^ by fluorescence microscopy. While mGFP-CAPRIN1 displayed a diffuse signal, mGFP-CAPRIN1^P512L^ formed small agglomerates, confirming the increased aggregation propensity seen in our cell models (Fig. 6g). Since CAPRIN1 is an RBP and recent studies demonstrated the pivotal role of RNA in the modulation of protein aggregation [63, 64], we next incubated the purified proteins with RNA. Strikingly, while mGFP-CAPRIN1 remained soluble, mGFP-CAPRIN1^P512L^ formed large, microscopically visible aggregates (Fig. 6g). This effect was independent of the RNA type, and all RNA types tested caused aggregation of mGFP-CAPRIN1^P512L^ (Fig. S5b). Since the association of RBPs with nucleic acids is often driven and stabilized through electrostatic interactions, we increased the salinity after complex formation to distinguish weaker (reversible) from stronger (irreversible, indicative of aggregates) interactions. Increasing the salinity reduced the degree of aggregation only to some extent, and the addition of RNase A did not dissolve the aggregates (Fig. 6g). This suggests that CAPRIN1^P512L^ misfolding might be triggered by RNA, but that RNA is not necessary for aggregate persistence. Consistent with this, our FISH analysis in CAPRIN1^P512L^ transfected cells showed that the formed aggregates do not contain polyA RNA (Fig. S5c).

Taken together, these data indicate that the Pro512Leu mutation alters the dynamics of binding to RNA which might influence the aggregation propensity of CAPRIN1.

## Discussion

To date, *CAPRIN1* has been associated with two conditions: increased *CAPRIN1* expression has been connected to certain cancers [65], while its reduction has been linked with autism spectrum disorders and speech delay [14–17]. In contrast, we identify the recurrent de novo CAPRIN1^P512L^ mutation in two independent individuals with early onset progressive ataxia and intellectual disability, which increases the protein propensity to aggregate and causes electrophysiological alterations in iPSC-derived neurons.

Strikingly, both affected individuals carry the identical de novo c.1535C > T variant, an event which is per se highly unlikely to occur by chance (Note S2). This variant is not reported in gnomAD, where *CAPRIN1* constraint metrics indicate that the gene has a reduced tolerance for missense mutations (*Z* = 1.69; *o*/*e*: 0.76 (95% CI 0.69–0.84)) and missense SNVs in the ± 100 bp range from the variant have all very rare frequencies (< 0.0001).

The P512L substitution affects the highly conserved proline residue exchanging a secondary structure breaker for a non-polar, aliphatic leucine (Fig. 1d and e) [65]. Although this amino acid substitution affects a residue close to CAPRIN1 PrLD (residues 537–709) and its surrounding region is enriched for residues found in prion-like domains, such as serine and glutamine, the proline-surrounding sequence is highly conserved. This suggests that this region is not disordered but adopts a specific fold that is most probably disrupted by the introduction of the leucine residue. In particular, the fact that the P512L substitution lead to an increase in the hydrodynamic radius of the protein, suggests that this proline is in the *cis* configuration in the wild-type protein and generates a kink in the polypeptide chain [66]. We hypothesize that this kink is absent in CAPRIN1^P512L^, causing the observed extended conformation. Moreover, due to the distance of this residue from potential post-translational modifications sites, this substitution is unlikely to influence them [67].

Our data suggest that the leucine substitution renders the protein prone to misfolding and aggregation, which is accelerated by the presence of RNA. Therefore, it is highly likely that the P512L acts in a gain-of-function manner. Furthermore, the gain-of-function model is in accordance with the increasing evidence that, conversely, CAPRIN1 reduction (e.g. haploinsufficiency) causes a different phenotype, characterized by language impairment, attention-deficit/hyperactivity disorder.

deficit hyperactivity disorder (ADHD) and ASD [14–17]. This suggests an intriguing parallel between CAPRIN1 and FMR1: FMR1 loss-of-function mutations are linked to Fragile X syndrome, a neurodevelopmental disorder characterized by intellectual disability due to hypermethylation of long CGG repeats (> 200) [68], while FMR1 gain-of-function mutations are linked to FXTAS, a neurodegenerative disorder where shorter CGG expansions (50–200) lead to increase in FMR1 expression [39, 69]. An important difference between the CAPRIN1 neurodegenerative disorder and FXTAS is the different disease onset: while the former occurs during childhood, the latter affects individuals older than 50 years of age [39]. This suggests that the protein quality control machinery is unable to control the aggregation properties of CAPRIN1^P512L^ and it could be related to the importance of the other proteins sequestered in the aggregates.

Intriguingly, we observed that CAPRIN1^P512L^ inclusions contain ATXN2 and GEMIN5. This is interesting because ATXN2 polyQ repeats cause autosomal dominant SCA2 (≥ 33 repeats) or increase ALS risk (31–32 repeats) through neuronal ATXN2 aggregation [10] and the affected individuals show both progressive ataxia and muscle weakness and atrophy. On the other side, biallelic mutations in GEMIN5 have recently been linked to early-onset neurodevelopmental delay and ataxia [38]. This suggests that sequestration of ATXN2 and GEMIN5 in CAPRIN1^P512L^ inclusions could at least in part be responsible for the observed gain-of-function phenotypes. In fact, CAPRIN1 and many other stress granule proteins are embedded in a dense network of protein–protein interactions even before they assemble into stress granules [70–72]. The fact that mutant CAPRIN1 is in a near-native state, suggests that many of these interactions will remain intact during misfolding and aggregation. Accordingly, CAPRIN1 aggregation may inactivate many associated proteins, providing an explanation for the severity of the disease phenotype.

Given the importance of the cell-specific environment in investigating a phenotype [25], we prioritized a neuronal model over patient-derived cell lines. Therefore, we generated the heterozygous CAPRIN1^WT/P512L^ and the homozygous CAPRIN1^P512L/P512L^ isogenic iPSC lines using CRISPR/Cas9 technology and differentiated them in cortical neurons, since both affected individuals suffered also from intellectual disability and even showed cortical atrophy and to avoid the considerable limitations of iPSC-derived cerebellar neurons, such as low cellular yield, need of co-culture with mice-derived progenitors or long differentiation duration of the available protocols [73]. At neuronal maturation (D36), we did not observe marked differences in differentiation efficiency (Fig. S2b and S2c), which would indicate a correct neuronal maturation and confirm the neurodegenerative nature of the disorder. We did not observe the formation of CAPRIN1 aggregates, even after proteasomal inhibition in both iPSCs and iPSC-derived neurons (Fig. S3a, S3b and S4a). One possible justification is a caveat of the disease model per se: iPSC-derived neurons usually lack the maturity of postnatally differentiated neurons and this acquires particular relevance when investigating a neurodegenerative disorder, where neuronal ageing plays a crucial role in the development of a phenotype [58]. In fact, iPSC-derived neurons used to study diseases characterized by protein aggregation like Alzheimer’s disease, Parkinson’s disease or Huntington’s disease mostly failed to detect amyloid beta, α-synuclein or HTT aggregation, respectively [56–58]. Based on the characterization of the iPSC-derived neurons conducted in the protocol’s original paper [25], it is reasonable to expect that the iPSC-derived neurons used in this study are not mature enough. Indeed, studies have shown that bypassing the iPSC state through direct reprogramming of somatic cells maintained aging hallmarks and recapitulated some disease phenotypes that are not present in iPSC-derived neurons [74]. This alternative approach was not pursued because of reproducibility concerns. However, it is important to note that iPSC-derived neurons could exhibit disease-specific alterations even in the absence of overt protein aggregation [58]. Interestingly, the activity of the iPSC-derived neurons decreases with the number of mutated CAPRIN1 alleles: while the spike rate and burst number of CAPRIN1^WT/P512L^ neurons is initially comparable to the one of CAPRIN1^WT/WT^ neurons, that of CAPRIN1^P512L/P512L^ neurons is always low: this suggests that pathophysiological changes in the neurons precede the formation of aggregates. This is in agreement with many NDs characterized by protein aggregation, where alterations in several cellular processes are found in neurons without inclusions, even in animal models [59, 75]. In CAPRIN1^WT/P512L^ neurons, the mutation causes an increase in the SG formation and a reduction in their resolution, in line with disease-linked mutations in other ND-related genes (*TARDBP*, *FUS* or *C9ORF72*) [60–62]. However, the homozygous mutation does not cause an increase in their formation, but even a slightly faster resolution. A possible explanation of this phenomenon is that the increased aggregation propensity of CAPRIN1^P512L^ might be counterbalanced by its reduced affinity for RNA. In homozygous CAPRIN1^P512L^ neurons, the protein’s low affinity for RNA tends to hinder the formation of SGs, compensating its increased aggregation. By contrast, in heterozygous neurons, the aggregation-prone but low-RNA-binding CAPRIN1^P512L^ can still form dimers with the wild-type CAPRIN1, and its aggregation propensity might increase SGs formation. These data suggest differences in the assembly of SGs that could be due altered RNA binding affinity, but whether SGs promote the formation of pathological CAPRIN aggregates or whether CAPRIN1 aggregates independently of SGs remains to be investigated.

Indeed, the pivotal role of protein-RNA interactions in aggregation is becoming increasingly clear: while for some mutations of ALS-related genes, such as *TARDBP*, *FUS*, *HNRNPA2B1* and *HNRNPA1*, the purified mutated proteins alone were often sufficient to enhance its aggregation propensity [20, 40–42], recent studies demonstrated the RNA ability to hinder or promote aggregation [63, 64, 76]. For example, RNA is able to antagonize TARDBP aggregation and disease-associated mutations would promote aberrant phase transitions of RNA-deficient TARDBP proteins [77]. Conversely, RNA increases the aggregation propensity of CAPRIN1^P512L^ and RNA removal does not revert the conformational change: this indicates that the misfolding might be irreversible. Interestingly, polyG RNA is able to trigger not only CAPRIN1^P512L^ aggregation but the one of CAPRIN1 too. A possible explanation for this phenomenon could be the intrinsic ability of polyG RNA to undergo phase separation due to the formation of G-quadruplex structures [78].

In conclusion, we identify with the P512L mutation a highly critical domain in CAPRIN1, the alteration of which associates with early-onset ataxia and intellectual disability, thereby associating another PrLD-containing protein to a novel neurodegenerative disorder. Moreover, we provide further evidence for the pivotal role of protein-RNA interactions in the assembly of aggregates.

## Supplementary Information

Below is the link to the electronic supplementary material.

Supplementary file1 (DOCX 5434 KB)

## Data Availability

The exome data of family A are stored in the EGA database under the access numbers EGAN00001366922, EGAN00001366923, EGAN00001366924. Request should be addressed to the NeurOmics data sharing committee. The exome data for family B can be made available upon reasonable request.

## Abbreviations

ADHS: Attention deficite/hyperactivity disorder
ALS: Amyotrophic lateral sclerosis
ASD: Autism spectrum disorder
CAPRIN1: Cell cycle associated protein 1
BSA: Bovine serum albumin
crRNA: CRISPR RNA
DLS: Dynamic light scattering
FBS: Fetal bovine serum
FCS: Fluorescence correlation spectroscopy
FTLD: Frontotemporal lobar degeneration
FXTAS: Fragile X tremor/ataxia syndrome
HD: Hungtington’s disease
iPSC: Induced pluripotent stem cell
gRNA: Guide RNA
LBD: Lewy bodies dementia
MEA: Microelectrode array
MRI: Magnetic resonance imaging
nanoDSF: Nano-differential scanning fluorimetry
ND: Neurodegenerative disorder
PD: Parkinson’s disease
PEI: Polyethylenimine
PFA: Paraformaldehyde
PQC: Protein quality control
PrLD: Prion-like domain
PBS-T: PBS + 0,2%tween20
RBP: RNA-binding protein
SCA2: Spinocerebellar ataxia 2
SG: Stress Granule
SSEP: Somatosensory evoked potential
ssODN: Single-stranded oligodeoxynucleotides
ssRNA: Single stranded RNA
UPS: Ubiquitin–proteasome system
TBS-T: TBS + 0.2%tween20
tracrRNA: Transactivating CRISPR RNA
UTR: Untranslated region
WES: Whole exome sequencing
WT: Wild-type
